# RNA polymerase I–Rrn3 complex at 4.8 Å resolution

**DOI:** 10.1038/ncomms12129

**Published:** 2016-07-15

**Authors:** Christoph Engel, Jürgen Plitzko, Patrick Cramer

**Affiliations:** 1 Max Planck Institute for Biophysical Chemistry, Department of Molecular Biology, Am Fassberg 11, 37077 Göttingen, Germany; 2 Max Planck Institute for Biochemistry, Department for Molecular Structural Biology, Am Klopferspitz 18, 82152 Martinsried, Germany

## Abstract

Transcription of ribosomal DNA by RNA polymerase I (Pol I) requires the initiation factor Rrn3. Here we report the cryo-EM structure of the Pol I–Rrn3 complex at 4.8 Å resolution. The structure reveals how Rrn3 binding converts an inactive Pol I dimer into an initiation-competent monomeric complex and provides insights into the mechanisms of Pol I-specific initiation and regulation.

## Abstract (web summary)

RNA polymerase I is the central enzyme that synthesizes ribosomal RNA in eukaryotic cells, and its regulation underlies cell growth. Here the authors present a high-resolution cryo-EM structure of the Pol I-Rrn3 complex that explains how Rrn3 specifically recognizes Pol I to form an initiation competent complex.

## Graphical abstract

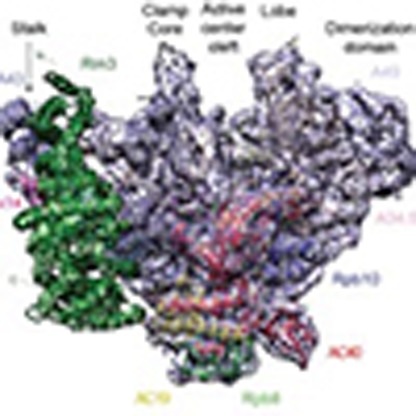

Transcription initiation by RNA polymerase (Pol) I is tightly regulated and requires the Pol I-specific factor Rrn3 in the yeast *Saccharomyces cerevisiae*^1^,^2^,^3^ and the Rrn3 counterpart TIF-IA in mammals^4^. In cells, only a fraction of Pol I enzymes are bound by Rrn3 and these Pol I–Rrn3 complexes can efficiently initiate transcription^2^. Previous structural work on the Pol I transcription system provided crystal structures of yeast Rrn3 (ref. 5) and the 14-subunit Pol I enzyme^6^,^7^,^8^. Evidence for the location of Rrn3 on the Pol I surface was also obtained^5^,^9^,^10^, but a detailed structure of the Pol I–Rrn3 complex is lacking.

The Pol I structure revealed an inactive, dimeric form of the enzyme with two prominent Pol I-specific protein elements called the expander and the connector. The expander occupied the active centre and was predicted to interfere with DNA binding. The connector mediates Pol I dimerization by binding the clamp of the adjacent polymerase within the Pol I dimer. It was proposed that the connector and expander must detach to activate Pol I and enable transcription initiation^6^. In particular, the connector must be displaced to enable formation of an initiation-competent Pol I–Rrn3 complex. To test this model, we determined the structure of the Pol I–Rrn3 complex.

## Results

### Formation and EM analysis of the Pol I–Rrn3 complex

We purified endogenous yeast Pol I (refs 6, 11), recombinant Rrn3 (ref. 5) and another Pol I-specific initiation factor, the three-subunit core factor^12^,^13^,^14^,^15^,^16^, and assembled complexes that contained all 18 polypeptides (Methods). These complexes were crosslinked and subjected to cryo-electron microscopy (cryo-EM) analysis essentially as described^17^ (Methods; Supplementary Fig. 1). A data set of 1,174 micrographs was collected on a Titan Krios equipped with a Gatan K2 direct electron detector. A total of 258,010 particle images were extracted, processed and subjected to classification. This revealed that the complex had largely dissociated under cryo-EM conditions. The largest fraction of particles were free Pol I enzymes that either lacked the A49/A34.5 subcomplex or displayed a flexible clamp-stalk region and hence did not yield high-quality reconstructions. A fraction of 63,445 particles corresponded to intact Pol I–Rrn3 complexes that enabled structure determination with the use of frame alignment and movie processing in RELION (Methods).

**Figure 1:**
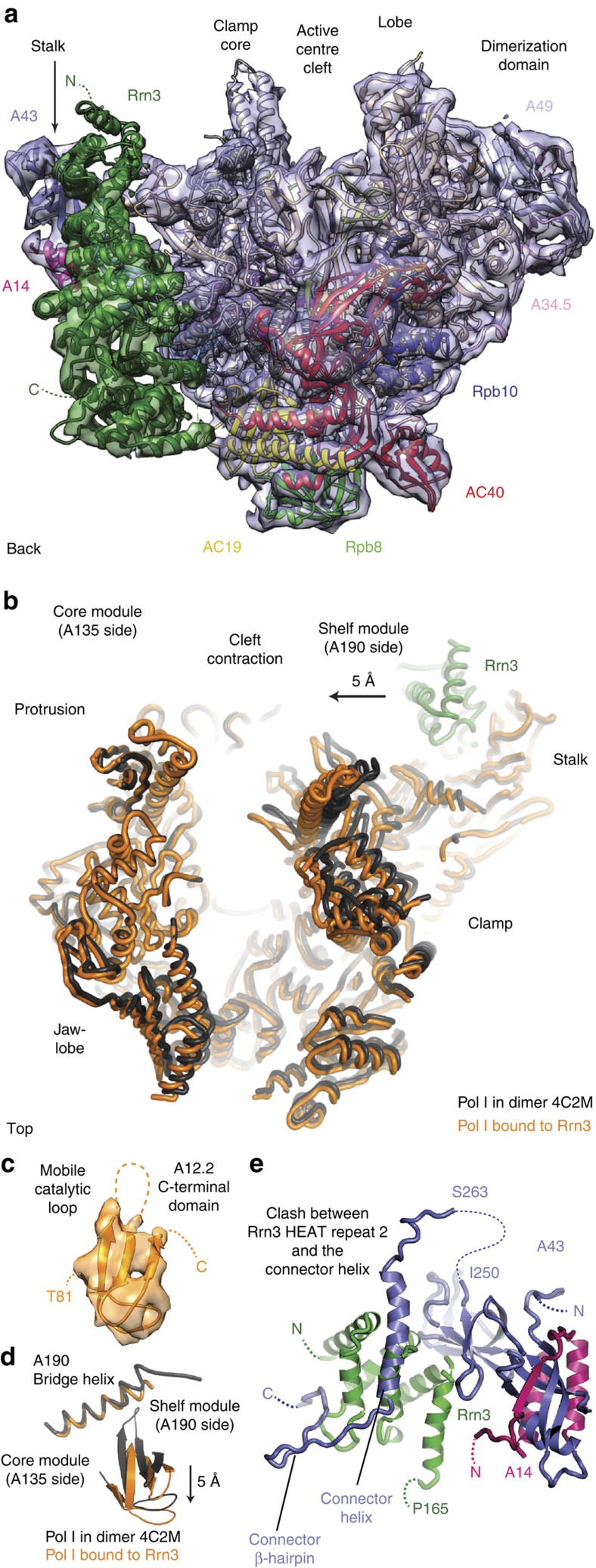
Cryo-EM structure of the Pol I–Rrn3 complex at 4.8 Å resolution. (**a**) Overview of the cryo-EM density (mesh) with fitted atomic structures (ribbon models) of Pol I (subunits in previously used colors^6^) and Rrn3 (green). The view is from the back^33^. (**b**) Partial cleft closure observed by superposition of the expanded dimeric Pol I crystal structure (black, PDB-code 4C2M) and the partially contracted, Rrn3-bound form (orange). The A135 subunits were superimposed. (**c**) The A12.2 C-terminal domain shows cryo-EM density and is well defined in the Pol I–Rrn3 complex structure, although the catalytic loop is mobile. (**d**) Rotation of the A12.2 C-terminal domain compared with the position observed in the free Pol I structure. The nearby bridge helix is partially rewound. (**e**) Superposition of the free Pol I crystal structure onto the Pol I–Rrn3 complex structure reveals that the connector (space filling, blue) would clash with the three N-terminal HEAT repeats of Rrn3 (green).

This resulted in a cryo-EM single-particle reconstruction of the Pol I–Rrn3 complex at 4.8 Å resolution. The cryo-EM map of the Pol I–Rrn3 complex was of great quality, clearly revealing secondary structure elements (Fig. 1a). The density allowed us to unambiguously place the crystal structures of Pol I (ref. 6) and Rrn3 (ref. 5), and to fit protein domains into densities as rigid bodies (Methods; Supplementary Fig. 1). The resulting Pol I–Rrn3 pseudo-atomic model revealed that Rrn3 binds between the Pol I stalk subcomplex A14/A43 and the AC40/AC19 heterodimer (Fig. 1a) as predicted from crosslinking^5^, and is generally consistent with early topological EM work^9^.

### Rrn3 stabilizes monomeric Pol I

When the Pol I crystal structure is compared with the cryo-EM structure of the Pol I–Rrn3 complex, several changes in the enzyme are observed. The previously expanded polymerase cleft is partially contracted, leading to a narrowing of the active centre cleft by 5 Å near its upper rims (Fig. 1b). The expander is apparently displaced, and the clamp is partially closed, albeit not as much as would be expected in a transcribing complex. The C-terminal domain of the Pol I subunit A12.2, which reached the Pol I active site via the pore in the structure of the dimeric enzyme, is rotated and slightly withdrawn from the active site, and its catalytic loop is mobile (Fig. 1c,d). Movement of the A12.2 C-terminal domain is accompanied by a partial rewinding of the previously unwound central region of the bridge helix (Supplementary Fig. 2). In addition, peripheral subcomplexes are slightly shifted (Supplementary Fig. 3). and the connector is displaced as predicted. Modelling shows that its former position would clash with Rrn3 on the Pol I surface (Fig. 1e). These structural changes explain how Rrn3 binding to Pol I stabilizes a monomeric Pol I–Rrn3 complex^5^ and indicate that Rrn3 induces a Pol I conformation that is closer to that expected for an active, transcribing enzyme.

### Pol I-specific interfaces mediate Rrn3 binding

**Figure 2:**
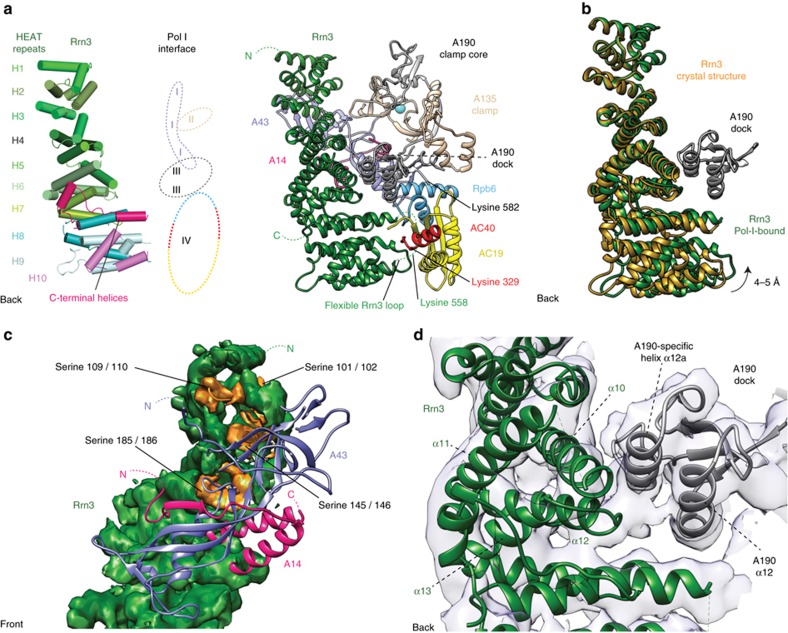
Details of the Pol I–Rrn3 interface. (**a**) Four interfaces between Rrn3 and Pol I. Schematic representation (left) and cartoon model (right) of Rrn3 HEAT repeats and their Pol I—interaction interfaces I–IV are labeled (see text). The flexible Rrn3 loop 551–580, which contains lysine 558, and its crosslinked interaction partners K329 (in AC40) and K582 (in A190) are indicated^5^. The view is from the back of polymerase^33^. (**b**) Bending of Rrn3 towards Pol I upon Pol I–Rrn3 complex formation. A hinge between the N- and C-terminal parts of Rrn3 enables rotation of the C-terminal region towards Pol I and tight interaction with the AC40/AC19 heterodimer. (**c**) Interface I viewed from the front. All eight serine residues (orange) that compose the ‘serine patch' of Rrn3 (ref. 5; space filling, green) are located in the interface and face the stalk. (**d**) Interface III viewed from the back. A second interface is constructed between the A190 dock domain and the Rrn3 helices α10 and α12 as well as rearranged loop between α12 and α13. The Rrn3-interacting A190 helix α12a and the subsequent loop are specific to Pol I.

The complex structure also shows that Rrn3 forms four interfaces with Pol I (Fig. 2a). To achieve an intimate interaction, the superhelical HEAT-repeat fold of Rrn3 is slightly bent towards the Pol I surface (Fig. 2b). The first interface is formed between Rrn3 HEAT repeats H2–H4 and the Pol I stalk subunit A43 (Fig. 2c). This interface contains a patch of serine residues in Rrn3 that are required for normal cell growth and Pol I promoter recruitment *in vivo*^5^. Phosphorylation of this serine patch represses mammalian Pol I transcription^18^,^19^ apparently because it prevents Pol I interaction with the Rrn3 counterpart TIF-IA. The interface also contains residues in A43 that get phosphorylated^20^,^21^ and may promote Rrn3 binding.

The second interface is formed between the N-terminal residues of helix α6 in Rrn3 HEAT repeat 3 and the A135-specific insertion 1,118–1,122 in the Pol I clamp (Fig. 2a). The third interface is formed between Rrn3 HEAT repeats H5–H6 and the Pol I A190 dock domain (Fig. 2d). The corresponding dock domain in Pol II binds the Pol II-specific initiation factors TFIIB and Mediator^17^,^22^. Rrn3 binding preserves a cavity on top of the dock domain, a location where the zinc ribbon domain in TFIIB binds Pol II (ref. 23) and where the corresponding domain in the core factor subunit Rrn7 (refs 24, 25) likely binds. The forth interface is formed between the C-terminal Rrn3 ‘interaction' loop α20–α21 (residues 552–580) and the C-terminal part of Pol I subunit AC40 (residues 330–335), AC19 residues 50–59, which form a loop that changes conformation, and the Rpb6 loop 112–114. This location of the Rrn3 α20–α21 loop furthermore explains the two previously reported protein–protein crosslinks connecting Rrn3 residue K558 to residues K582 and K329 in Pol I subunits A190 and AC40, respectively^5^.

## Discussion

Taken together, our results not only confirm previous predictions on the location of Rrn3 on the Pol I surface. They additionally show that Pol I-specific elements recognize Rrn3 and provide details on the Pol I–Rrn3 interaction. The results support a previous structural model for Pol I activation^6^. Briefly, Pol I exists in an equilibrium between inactive Pol I dimers and activatable monomers^6^. Monomers can be withdrawn from the equilibrium by stable association with Rrn3, which prevents Pol I dimerization, thereby rendering Pol I initiation-competent. Our Pol I–Rrn3 structure now provides detailed insights into how this transition is accomplished. First, release of the connector liberates the Pol I surface required for Rrn3 binding. Second, Rrn3 binding sterically interferes with connector re-association and thus stabilizes Pol I in an initiation-competent monomeric form. Third, Rrn3 binding leads to a partial contraction of the Pol I cleft that apparently releases the expander and liberates the DNA template-binding site, which is a prerequisite for DNA loading and transcription initiation. Fourth, the A12.2 C-terminal domain is slightly repositioned and its catalytic loop becomes mobile, likely to prevent RNA cleavage during initiation. Our results represent the first step towards a mechanistic analysis of Pol I initiation, which in the future must address the question how promoter DNA is specifically recognized and loaded into the active centre cleft.

## Methods

### Sample preparation

Endogenous Pol I and recombinant Rrn3 were expressed and purified as described^5^,^6^. Recombinant core factor was expressed in BL21DE3(RIL) cells^12^ and purified essentially as published^13^. Purified Pol I was incubated with a fivefold access of Rrn3 and CF, and incubated overnight at 4 °C. Excess factors were removed by size-exclusion chromatography with a Superose 6 10/300 GL column (GE Healthcare) in buffer A (150 mM sodium chloride, 5 mM HEPES (pH 7.8), 1 mM magnesium chloride, 10 μM zinc chloride, 5 mM dithiothreitol). The Pol I–Rrn3–CF complex was crosslinked with 1 mM BS3 (Sigma) at 30 °C for 30 min and the reaction mixture was quenched with 50 mM ammonium hydrogen carbonate for 20 min at 25 °C. Excess BS3 was removed by subsequent size-exclusion chromatography using a Superose 6 3.2/300 GL column (GE Healthcare) in buffer A.

### Electron microscopy

A single 0.05-ml peak fraction was diluted to a concentration of 0.10 mg ml^−1^ for grid preparation. R2/1 holey carbon grids (Quantifoil) were glow-discharged for 15 s and 5 μl of sample was incubated for 10 s at 4 °C and 100% humidity before blotting and plunging into liquid ethane with a Vitrobot Mark IV (FEI). Cryo-EM data was acquired on a FEI Titan Krios operated in EFTEM mode at 300 keV. Data were collected with a K2 Summit direct detector (Gatan) and the TOM toolbox^26^. A total of 1,174 movies were acquired with a defocus range from –0.8 to –3.6 μm at a nominal magnification of × 37,000 or 1.35 Å per physical pixel. The camera was operated in ‘super-resolution' mode (0.675 Å per pixel) and binned to 1.35 Å per pixel, with a total exposure time of 9.9 s split into 33 frames, at a dose rate of ∼4 e^–^ per pixel per second and total dose of 40 e^–^ Å^–2^ per movie.

### Image processing

Movies were aligned as described^17^, without partitioning into quadrants. CTF (Contrast Transfer Function) estimation was carried out with CTFFIND4 (ref. 27) and further processing was performed with RELION 1.3 (including CTF correction)^28^. An initial data set of 9,010 particles was semi-automatically picked using EMAN2 (ref. 29) with a box size of 320 Å^2^. Reference-free two-dimensional classes were calculated and five classes were low-pass filtered to 25 Å to serve as templates for automated picking. From the 1,174 micrographs of good quality, a total of 484,806 particles were auto-picked. Two-dimensional classification, *Z*-score-dependent sorting and manual screening led to a data set of 258,010 particles that were subjected to further analysis. A Pol I monomer (PDB code 4C2M) filtered to 40-Å resolution was used for an initial three-dimensional (3D) refinement. Particle polishing was performed using the movie frames 3–20 to diminish the effects of beam-induced motion (frame 1 and 2) and radiation damage (frame 21–33). Polished particles were subjected to multiple rounds of 3D classification with and without alignment to remove (1) free Pol I suffering from conformational heterogeneity, caused by movements of the clamp and the stalk, (2) Pol I lacking the A49/A34.5 dimerization domain and (3) a very low number of particles containing CF. The remaining 63,445 particles showed a strong density for the polymerase core and all peripheral domains including the A190 clamp, the A49/A34.5 dimerization domain and the A14/A43 stalk as well as Rrn3. A mask encompassing Pol I and Rrn3 was calculated using RELION and used in 3D refinement to yield a 4.8 Å reconstruction with evenly distributed particles (Supplementary Fig. 1). Resolution is based on the gold-standard FSC (0.143 criterion)^30^ and temperature factors were automatically determined and applied in RELION (−165 Å^2^ for the final reconstruction).

### Structure modelling

At the nominal resolution of 4.8 Å, we derive a pseudo-atomic model based on the published crystal structures, but refrained from detailed modelling at the level of amino-acid residues. A model of a Pol I monomer lacking the expander and the connector was constructed from the PDB entry 4C2M using COOT^31^ and placed into the density using UCSF Chimera^32^. Previously defined domains of Pol I (ref. 6) were rigid body-fitted in real space using COOT^31^. A Rrn3 monomer (PDB 3TJ1) was also fitted to the density with USCF Chimera and its HEAT repeats or, if required, single helices were adjusted slightly with COOT. Geometric parameters of residues located in connections between shifted domains were regularized applying standard geometrical restraints in COOT. Figures were prepared with UCSF Chimera^32^ or PyMOL (www.pymol.org) and graphs were calculated and visualized with GraphPad Prism (www.graphpad.com).

### Data availability

The cryo-EM density has been deposited in the Electron Microscopy Data Bank under accession code EMD-3439 and coordinates of the Pol I–Rrn3 model have been deposited with the Protein Data Bank under accession code 5G5L. The authors declare that all data supporting the findings of this study are available within the article and its Supplementary Information files.

## Additional information

**How to cite this article:** Engel, C. *et al*. RNA polymerase I–Rrn3 complex at 4.8 Å resolution. *Nat. Commun.* 7:12129 doi: 10.1038/ncomms12129 (2016).

## Supplementary Material

Supplementary Information Supplementary Figures 1-3
