# Supplementary material for: RNA polymerase I–Rrn3 complex at 4.8 Å resolution
Source: Nat Commun. 2016 Jul 15;7:12129. doi: 10.1038/ncomms12129 (PMC4947163; doi:10.1038/ncomms12129)
Supplement: Supplementary Information — Supplementary Figures 1-3 [file ncomms12129-s1.pdf]

SUPPLEMENTARY INFORMATION

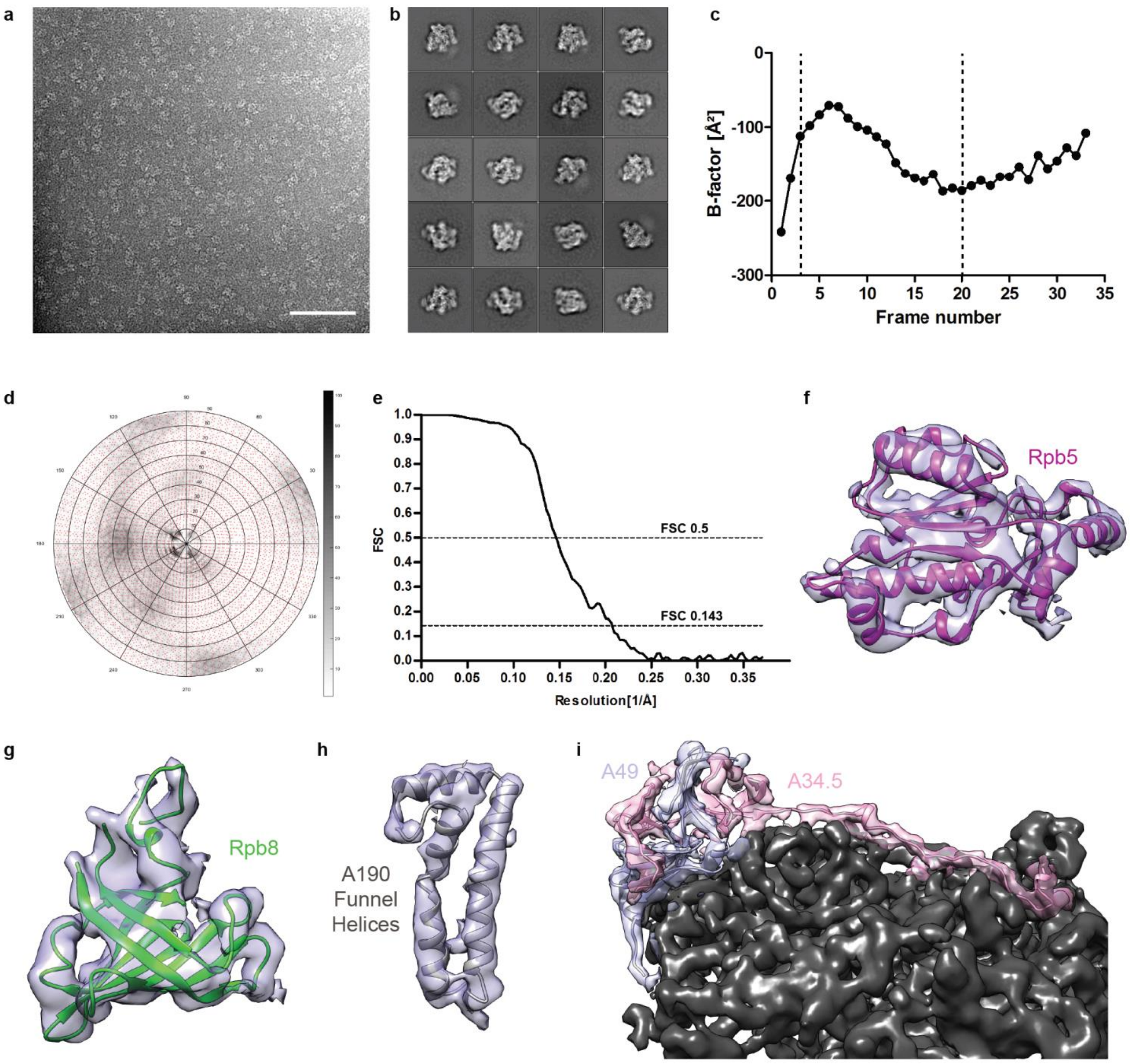

Supplementary Figure 1. Quality of the Pol I - Rrn3 cryo-EM reconstruction.

(a) Exemplary micrograph of the analyzed cryo-EM dataset. Scale bar is 100 nm. (b) Representative 2D class averages of the particles used for the Pol I - Rrn3 cryo-EM reconstruction. (c) B-factor plot for the frames used in particle polishing. Frames 1, 2 and 21-33 were discarded. (d) Angular distribution of single particle orientations used in cryo-EM reconstruction of the Pol I-Rrn3 complex. Shades indicate the number of particles assigned to a view; red dots indicate represented views. (e) FSC plot for the Pol I - Rrn3 reconstruction. 0.143 and 0.5 FSC limits indicated. The first data point after phase randomization is omitted. (f)-(i) Example cryo-EM densities of Pol I domains indicate the high overall quality of the reconstruction (common polymerase subunits Rpb5 (f) and Rpb8 (g), A190 funnel domain (h) and the A49/A34.5 sub-complex including the dimerization domain and the A34.5 C-terminal tail (i)).

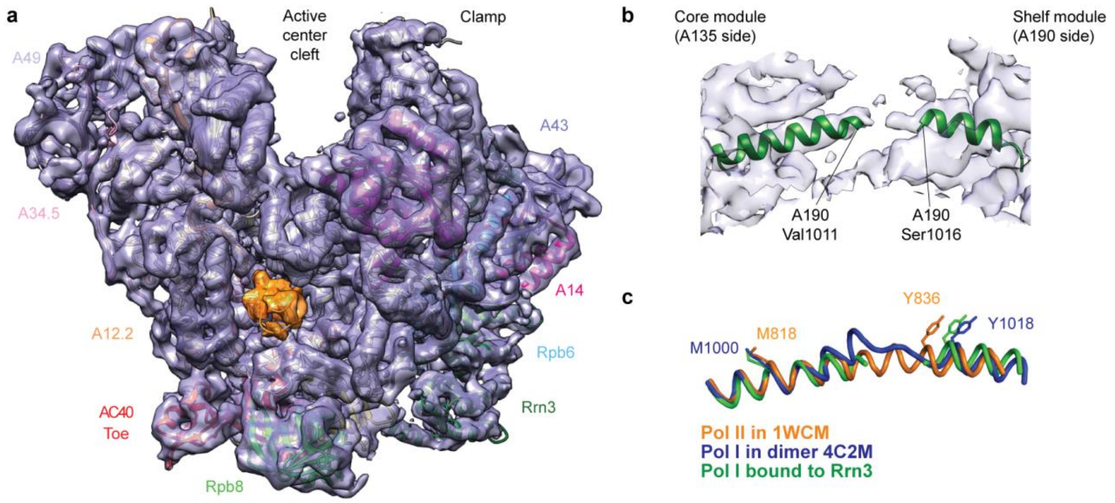

Supplementary Figure 2. A12.2 and the A190 bridge helix

(a) Location and density of the C-terminal domain of A12.2 (orange). (b) Compared to the free Pol I dimeric structure, the bridge helix partially rewinds but still displays no density at its center. Therefore, it is not entirely helical in the Rrn3-bound form and likely contains a remaining unwound central region. (c) Comparison of bridge helices between Pol II, dimeric Pol I and the Pol I-Rrn3 complex.

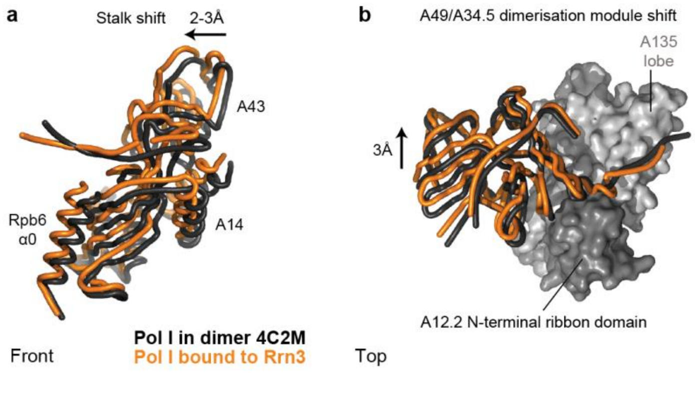

Supplementary Figure 3. Movements of Pol I sub-complexes upon Rrn3 binding.

(a) The A14/A43 stalk is shifted towards the top of Pol I by up to 3 Å. (b) The dimerization domain of the A49/A34.5 sub-complex is shifted towards the back of Pol I whereas the A135 lobe and A12.2 N-terminal ribbon domains remain mostly immobile (displayed in space-filling light and dark grey, respectively).
